# Poster 2001: Generation a hypoallergenic profilin from amaranthus palmeri pollen

**DOI:** 10.1186/1939-4551-7-S1-P18

**Published:** 2014-02-03

**Authors:** Cesar Manuel Landa, César Augusto Sandino Reyes

**Affiliations:** 1 Escuela Nacional de Médicina y Homeopatia, Instituto Politecnico Nacional, México Distrito Federal, Mexico

## Background

Amaranthus pollen grains are known to be highly allergenic and a potential cause of respiratory allergic diseases in many countries. *Amaranthus palmeri* pollen is an important allergenic source in Mexico; however in our country, there is not reported data about the prevalence of these pollens. The first allergenic profilin was identified from birch pollen allergen (Bet v 2) in 1991. Many studies have been described that patients who developed allergies against profilin from pollen commonly presented cross-reactivity against to vegetable food profilin, in particular to different fruits as nuts, melon, orange, and peach, or to vegetables as zucchini, celery, tomatoes and carrots or also latex.

The aim of the study is to clone the profilin from *A. palmeri* pollen and obtain hypoallergenic profilin variant, this variant could be used as a therapeutic.

## Methods

cDNA from *A. palmeri* pollen profilins were amplified and cloned into the pJET1.2/blunting vector and subcloned into over expression vector. Profilins were purified by affinity chromatography using as ligand poly-L-proline coupled to sepharose. Profilins purified for natural source from *A. palmeri* pollen were named as naturals profilin (nAmpa) and recombinant proteins were named as rAmpa. In addition, we obtained the circular dichroism spectrum of four different isoforms. Profilins were analyzed by ELISA using serum allergenic patients from two groups. The first group was formed by patients with positive Skin prick tests (SPT) for *A. palmeri*pollen and second group was formed by allergic volunteers without SPT.

## Results

Eight profilins were cloned and cDNA sequencing revealed that seven of these are 393 bp encoding for 131 amino acid residues and the other cDNA is 399 bp encoding for 133 amino acid residues. These isoforms were named rAmpa ISO-1 to -8. rAmpa ISO-1, -4, -7 and -8 were expressed in *E. coli* as a soluble protein and purified at one step for affinity chromatographic in similar conditions that naturals profilin (nAmpa). The spectrums obtained from *A. palmeri* profilin (rAmpa) are similar to *Phleum pratense* natural profilin previously reported. One of the eight profilins was less recognized than others by serum from patients allergenic, this profilin could be used as a vaccine for allergenic patients to profilin.

## Conclusions

Profilin from *Amaranthus palmeri* pollen could be used as a treatment.

